# Surface Modification of Biomedical and Dental Implants and the Processes of Inflammation, Wound Healing and Bone Formation

**DOI:** 10.3390/ijms11010354

**Published:** 2010-01-25

**Authors:** Clark M. Stanford

**Affiliations:** Dows Institute for Dental Research, College of Dentistry, University of Iowa, IA 52242, USA; E-Mail: Clark-Stanford@uiowa.edu; Tel.: +1-319-335-7381; Fax: +1-319-335-8895

**Keywords:** implant, surfaces, wound healing, macrophage

## Abstract

Bone adaptation or integration of an implant is characterized by a series of biological reactions that start with bone turnover at the interface (a process of localized necrosis), followed by rapid repair. The wound healing response is guided by a complex activation of macrophages leading to tissue turnover and new osteoblast differentiation on the implant surface. The complex role of implant surface topography and impact on healing response plays a role in biological criteria that can guide the design and development of future tissue-implant surface interfaces.

## 1. Introduction

Endosseous dental implants have created a revolution in the routine approach to dental care for patients missing one or more teeth. The clinical success for this procedure occurs through a series of clinical and biological steps starting with initial primary stability provided by the amount, quality and distribution of bone within the proposed implant site [1]. Following placement of the dental implant a series of bone modeling and remodeling steps take place. Bone adaptation or integration of an implant is characterized by a series of biological reactions that start with bone turnover at the interface (a process of localized necrosis), followed by rapid repair [2]. The common clinical end point of this process is measured by a lack of signs and symptoms of aggressive chronic inflammation, a lack of mobility and a radiographic assessment of bone adapted to the interface [3,4]. While high success rates hold for certain anatomic regions, the bony response within the thin cortical plates and diminished cancellous bone characterizing the Lekhom and Zarb type IV bone is considerably less successful (e.g., 65–85%) with conventional machined surfaced implants [5]. These results may relate to both the minimally rough implant surfaces being used at the time of Lindh’s study and the patient population assessed. The response of trabecular bone to the mechanical environment is a critical factor, especially in regions of the jaw, such as the edentulous posterior maxillae, where the cortical thickness and/or local material properties are insufficient to withstand occlusal forces [2].

The long-term success of implant therapy is not just dependant on enhanced osseous stability. More recently, there is greater attention being addressed to the transmucosal dental implant or implant abutment interfaces. The mechanical and biological stability derived from the design and surfaces in this connective tissue and junctional epithelial environment are critical to maintaining a sufficient volume of connective tissue with minimal inflammatory infiltrate. Chronic inflammation in this transmucosal region can be influenced by the designs, materials or surface roughness leading to long-term tissue recession and even peri-implantitis years after the completion of tooth replacement therapy [6–13]. In order to increase the predictability of implant therapy, significant efforts have gone into development of implant biomaterials that hold the promise of improving clinical success. These technologies have evolved from simple modification of the oxide surface to precise nano-scale modification technologies that involve the formation of a uniform and consistent surface that leads to altered cellular response. Further, there are developing technologies to utilize changes in surface chemistry or even potentially biologics being added to the oxide surface to assist in stability of both the osseous and transmucosal environment.

The purpose of this review is to discuss some of the recent developments in titanium implant surface technology and to discuss the role in mediating macrophage biology in the wound healing site around the implant.

## 2. Implant Macro-Retentive Features

Implants used in the oral environment have one of three major types of macro-retentive features: screw threads (tapped or self tapping), solid body press-fit designs and/or sintered bead technologies. These approaches are designed to enhance initial implant stability and/or create large volumetric spaces for bone in-growth. An important biological principal of bone is that it responds favorably to compressive loading (without the presence of a ligament) but not to shear forces [14]. Therefore, screw thread implant designs have been adapted to achieve a compressive loading of the surrounding cortical or cancellous bone. Other thread designs focus on reducing the surrounding shear forces by reducing the height of the thread profile (reducing the contribution of any one thread) with an increase in the number of threads per unit area of the implant surface [15]. This has the additional benefit of increasing the strength of the implant body by increasing the amount of remaining wall thickness of the implant body [16]. Finally, orthopedic prosthesis (e.g., femoral stems, pelvic acetabular caps, knee prosthesis, *etc.*) have used various sintering technologies to create mesh or sintered beads as a surface for bone to grow in to. The application of this technology to dental implants has involved attempts to improve the success rate of short implants (<10 mm in length) [17]. This has been applied to a limited number of dental implant systems with very short implants [18–26].

## 3. Implant Micro-Retentive Features

Upon the placement of an implant into a surgical site, there is a cascade of molecular and cellular processes that provide for new bone growth and differentiation along the biomaterial surface. Following placement, the surrounding bone undergoes an initial necrosis, bone resorption and replacement, initially with a woven-like cell rich bone that is replaced through remodeling with mature haversian bone [27,28]. The goal of a number of current strategies is to provide an enhanced osseous stability through micro-surface mediated events. These strategies can be divided into those that attempt to enhance the in-migration of new bone (e.g., osteoconduction) through changes in surface topography (a.k.a., *surface roughness*), biological means to manipulate the type of cells that grow onto the surface and strategies to utilize the implant as a vehicle for local delivery of a bioactive coating (adhesion matrix or growth factor such as BMP-2) [29,30].

One means to improve implant success is through methods to increase the amount of bone contact along the body of the implant. While it may seem obvious that increased surface roughness of implants leads to greater success it is not clear what aspect of “roughness” is advantageous [31]. In dental implant design, it is assumed that a greater surface area (per unit of bulk metal surface) is an objective by various means to enhance the surface roughness of the implant surface. This enhanced surface area allows a greater area for load transfer of bone against the implant surface [32–35]. It should be clarified that surface roughness is often a poorly described characteristic [36]. Micromechanical features influence the process of secondary integration (bone growth, turnover and remodeling) [2]. One advantage of acid etching, a technique commonly used is to increase the roughness of the grit blasted surface creates the potential for a nanometer-scale topography on top of the macroscale roughness allowing bone to adapt to the surface under elevated shear forces [37,38]. Implant design features conventionally were thought to need surface pores or “pits” of 100 μm or greater in diameter for in growth of bone although clinically relevant surface roughness may actually be much finer (on the nanoscale level) [39].

## 4. Implant Wound Healing and the Potential Role of Surface Modification

Wound healing around a dental implant placed into a prepared osteotomy follows three stages of repair. Initial formation of a blood clot occurs through a biochemical activation followed by a cellular activation and finally a cellular response. As our understandings of these complex pathways are incomplete, there have been many approaches, especially *in vitro* systems, to tease apart the pathways. In doing this, it should be pointed out to the reader that most of our understanding of wound healing pathways extends from *in vitro* studies and this limits our understanding of both the redundancy of responses *in situ* as well as the potential validity of some observations made, *in vitro*.

These initial rapid changes during the surgical phase of implant therapy leads to activation of key biochemical pathways: the clotting system (fibrinogen to fibrin), complement activation, Kinin cascade activation (vascular dilation) and finally, plasmingen activation of plasmin. The adhesion of platelets to the assembled fibrin scaffold as well as adhesion to the surface topography of an implant surface leads to a process of platelet activation. Platelets are a rich source of locally released growth factors (e.g., PDGF, TGF-Beta, PDEGF, IGF-1) that accelerate the wound healing process though recruitment and differentiation of mesenchymal cells critical to establishing an osseous interface at the implant surface [40]. It is the interaction with the surface and serum proteins which appear to create the primary effect of implant surface topography [41]. Titanium surfaces that were modified though a controlled etching process have been shown to alter whole blood derived platelet adhesion and generated thrombin-antithrombin complexes [42]. Platelet activation has also been elevated on etched titanium surfaces. When platelet adhesion and activation was compared on machined versus blasted/etched titanium surfaces, *in vitro*, the smoother machined surfaces demonstrated higher adhesion of platelets but reduced activation while the rougher surfaces demonstrated reduced platelet adhesion but near 100% platelet degranulation [43].

During the initial remodeling steps, there are a number of immune cells that mediate early tissue (platelets, PMNs) followed by an in migration of phagocyte macrophages [44]. The complex and pluripotent role of macrophages has recently become engaged in biomaterials research not just as mediators of debris removal but also potentially playing a key role in mediating new bone formation on the implant surface [45,46]. Recent comprehensive reviews have articulated the complex role of macrophages and the conventional separation into the “classically activated macrophage pathway” (e.g., bacterial derived lipopolysaccaride or LPS) leading to activated Toll-like receptors relative to the “alternative macrophage activated pathway” (activation via IL-4, IL-13 and cell surface expression of mannose receptor (CD206) and Arginase-1 receptor) does not represent the current understanding of the phenotypic flexibility of this cell type. Mosser and Edwards suggest there is a continuum between these two cell types of macrophages and that wound healing is an important role of a subset of these cells [44]. An initial role for these cells is to remove the necrotic debris created by the drilling process and this material is laced with DNA fragments, histones, nuclear proteins and heat-shock proteins all of which leads to physiological changes in the macrophages, leading in turn to expression of cell surface proteins (CD135) production of cytokines and pro-inflammatory mediators though the NFKB pathway [44,47]. Dental implants are typically placed from a cortical surface of the dental alveolus though into the medullar cavity. It is interesting to note that when histological studies are performed on clinically healed implants, there is often bone contact exceeding 50% of the implant surface area extending along the portion of the device that passes though the medullar cavity whereas this does not occur in the absence of the medical device [48–51]. This allows rapid contact of the implant surface with marrow derived monocytes and may be one reason for the observation of extensive adhesion of macrophages to retrieved implant surfaces [45]. The following steps are not fully understood and may involve interactions of inflammatory mediators on activating these cells into tissue-macrophages. Activation of macrophages typically involves a combination of tumor-necrosis factor alpha (TNF-α) and interferon-γ (IFNγ) to promote a bactericidal phenotype (e.g., expression IL-1, IL-6 and IL-23) initially though an innate immune response [44]. From a biomaterials perspective, the influence on the wound healing capacity by macrophages may be strategic. These cells respond though both innate and adaptive responses which includes response to basophil and mast cell release of IL-4 eliciting a differentiation of macrophages into a wound healing pathway [52, 53]. Later, adaptive immune response occurs though T_H_2 helper cell and IL-10 expression leading to expression from an intermediate regulatory macrophage of IL4 and IL-13. This cytokine-regulated cellular recruitment, migration, proliferation and formation of an extracellular matrix on the implant surface can be influenced by this early population of macrophages. These cells express growth factors such as fibroblast growth factor (FGF-1, FGF-2, FGF-4), Transforming growth factors, epithelial growth factor as well as bone morphogenetic proteins (BMPs) [54,55]. The end result of this complex cascade is promotion of a wound healing process that includes angiogenesis. The development of an elaborate vascular network is an important part of the implant wounding healing process and may be elicited by the initial ischemia in the immediate wound site followed by the macrophage mediated release of bFGF, TNF-α and vascular endothelial growth factor (VEGF) [55–57].

The role of macrophages in implant topography has primarily focused more on polymer-based materials and the potential for inflammation in vascular-based devices. Macrophages are extensively involved in the complex process of aseptic necrosis in orthopedic applications which is primarily a response to polymer wear debris in the enclosed area of the implant site [58]. Dental implants typically have the part of the implant subject to wear being located within the oral cavity thus reducing the potential for macrophage-based activation to remove wear particles [59–61]. Recent work has demonstrated though that macrophage activation is influenced by surface topography of the biomaterial. Paul *et al*., (2008) demonstrated topographical control using a polyvinylididene fluoride (PVDF) surface created using laser ablation and showed that macrophage responded to macroscopic surface topography in which they adhere and spread but not to nanoscale surface features and most intriguing was that specifically the CD163 positive macrophages (those associated with the alternative activation cascade or M2 cells in the conventional classification of macrophages) leading to a suggestion that the inflammatory response to implant surfaces can downregulate the expression of pro-inflammatory cytokines and thus implant topography can play a role in early events in biocompatibility [62].

The subsequent formation of a mineralized matrix during osteogenesis and bone remodeling or during osseointegration of dental implants involves the recruitment of multipotent mesenchymal stem cells and the progressive differentiation of these cells into osteoblasts [63]. Osteoblast differentiation and skeletal formation during embryonic development is mediated by an essential transcription factor protein called core binding-factor-alpha (Cbfa1) or RUNX-2 [64]. Cbfa1 belongs to the Runt family of transcription factors [65], and regulates osteoblast differentiation and expression of bone extracellular matrix protein genes that encode for bone sialoprotein (BSP), Osteocalcin and Type I Collagen [66,67]. RUNX-2/Cbfa1 plays an essential role in osteogenesis, osteoblast matrix formation, chondrocyte differentiation, and bone resorption by osteoclasts [68], and could therefore be a downstream target of cellular events such as extracellular matrix adhesion-mediated signaling, changes in cell shape and responses to local paracrine environments. A second transcription factor, osterix, has been described and has been suggested to play a key role downstream of RUNX-2 in which its expression is necessary for the ongoing differentiation within in the osteogenic pathway (versus sifting to a chondrogenic pathway) [69]. In an *in vitro* study, Misaski *et al*., showed that human mesenchymal stems cells grown on titanium surfaces with a nanoetched topography had elevated RUNX-2 and Type I Collagen expression and specifically increased the expression of alkaline phosphatase, a key enzyme involved in the control of biomineralization at the implant surface [70]. Upregulation of osterix and BSP was noted on alumina coated titanium surfaces with a nanometer level topography, relative to surfaces with just micrometer-level surface features. Human mesenchymal stem cells were grown over a 28 day period and demonstrated specific response to the etched titanium surfaces [71–73].

The promotion of osteoblast attachment and differentiation has been evaluated on various implant surfaces using a variety of cell culture and animal models [2,74,75]. For instance, the expression of matrix related proteins such as alkaline phosphatase (an enzyme with a role in biomineralization) and Type I collagen were enhanced on sand blasted and acid etched cpTi surfaces [76] The mechanism by which topography influences osteoblast differentiation appears to be mediated by the protein kinase A and PL-A2 pathway [77], and by integrin mediated signaling pathways [74,78,79]. The topography also influences subsequent expression of osteoblast-mediated cytokines and growth factors. Osteosarcoma cells (MG-63) grown on roughened surfaces had increased TGF-Beta and IL-1beta [80] in which a prostaglandin mediated response was described leading to decreased proliferation on characterized rougher surfaces with an increase in cellular phenotypic markers of differentiation (ALP activity, osteocalcin). While these observations demonstrate cellular responses of osteoblasts to the matrix absorbed onto the surface of an implant, they do not provide information on the role the surface (and the resultant matrix topography) on initial platelet adhesion/activation events and subsequent alterations of osteoblast cell shape and differentiation. It may be possible that the implant topography may lead to enhanced differentiation of osteoblasts through alterations in transcriptional regulation or gene expression of key osteogenic factors as a result of changes in cell shape due to interaction with the implant surface microtopography [81].

## 5. Implant Micro-Retentive Features: Surface Roughness by Blasting/Etching

Various studies have also addressed the issue of surface roughness through various means of grit blasting followed by a surface etching or coating procedure. This has included titanium plasma spray (TPS) [35], abrasion (TiO_2_ blasting or use of soluble abrasives) combinations of blasting and etching (e.g., Al_2_O_3_ with H_2_SO_4_/HCL) [35], thin apatite coating [82] or sintered beads [83]. Commercially available roughened surfaces using the large grit blasted and acid etched surface (e.g., Straumann’s SLA surface) having both laboratory and clinical evidence of elevated success rates in areas of the posterior maxilla [84–88]. The role of the roughened surface is complex since the actual strength of bone contact against the titanium oxide surface is quite low (4 MPa or less); weak enough that without the surface topography (e.g., electropolished surfaces) little bone contact occurs [37]. A further modification of the SLA grit blasted and dual acid etched surface has recently been described [89]. In this procedure, the same surface roughening process occurs but there is a nitrogen atmosphere used to control the rate and formation of the oxide surface by attempting to reduce hydrocarbon contamination of the implant surface.

Various titanium surfaces have utilized surface roughness created either though a grit blasting and etching procedure or blasting of the surface alone by using tightly controlled conditions in order to obtain a pre-defined optimal surface topography. One such optimization criteria has been proposed [90,91]. This criteria suggests that an implant surface has an optimal balance between pore size on the surface (pore sizes of 1–5 μm diameter and 1–5 μm in depth) which optimizes the shear strength of the individual bone in-growth into anyone pit with the need to have as many “pits” on the surface as possible [15,34]. A further modification of the titanium dioxide grit blasted surface is then performed with a mild hydrofluoric acid etching to create surface pitting on the blasted surface. The optimization criteria calls for maintaining the macroroughness derived from the blasting process followed by the surface etching to influence the secondary osseointegration process (the process of wound healing following implant placement). Masaki *et al*. and Isa *et al*. used a human mesenchymal cell culture model and demonstrated there was a rapid increase in expression of key genes involved in the differentiation of bone unique to the fluoride modified and etched titanium surface that was not evident on blasted surface alone or a comparison group of large grit and dual acid etched surfaces [70,78]. In a follow-up study, Cooper *et al*. demonstrated that the same titanium surfaces also increases expression of bone adhesion and increased expression of bone sialoprotein, osteopontin and other bone-specific proteins critical to an accelerated bone adaptation to the implant surface [92].

Currently there are two main, but inter-related approaches being evaluated to enhance bone adaption to dental implant surfaces. Both approaches are designed to improve the adaptation of trabecular bone. The two approaches involve either the addition of biological mediators to the implant surface (e.g., cell adhesion or bioactive peptides, growth factors, *etc.*) or creation of reproducible nanoscale surface features.

The addition of bioactive peptides has a long and rich history in biomaterials research with strategies to covalently add either cell adhesion peptides or growth factors to the implant surface and thus use the implant as a local drug delivery device. The manner in which the fibrin scaffold is manipulated is one key to the future of implant surface technology [93–95]. Approaches to modifying the titanium oxide surface utilizes placement of various configurations of the commonly known recognition peptide sequences for cell adhesive integrins cell membrane associated matrix binding receptors (a tripeptide sequence of Arg-Gly-Asp or “RGD”) on the surface of an implant surface. This RGD sequence mediates cell adhesion and is present in a number of extracellular matrix proteins (e.g., fibrin, collagen, fibronectin, vitronectin, osteopontin and bone sialoprotein). Obviously, many mesenchymal cells possess integrin receptors and thus the adhesion to an RGD coated surface may be rather non-specific but a number of groups are attempting to regulate the type of cell adhesion that occurs by modulating the sequence of proteins in the linker region (the region of protein attaching the RGD sequence to the metal substrate) as well as exploring various means to attach adhesion sequences (e.g., repeated regions of RGD sequence) through covalent attachment to the surface of the implant [59,79,96–107]. Work has focused on determining the optimal density of RGD adhesion peptides on an experimental implant surface to elicit osteoblast growth and differentiation [99].

Osteoblastic cells interact both with the matrix, through the RGD dependent pathways, but also to the surface contours of the surface. This property of “contact guidance” is an important part of understanding the behavior of how pre-osteoblasts interact with the complex fibrin/platelet scaffold. There are a number of elegant *in vitro* and *in vivo* studies which document the importance of not just the roughness of an implant surface but the effect of the direction of the pattern (or epitaxis) [108–111]. These studies demonstrate that fibroblasts and osteoblasts are capable of recognizing repeated surface features (e.g., lines, grooves and other defects created in machining). Cells appear to align and grow in a directional pattern directed by the surface features of the substratum [112]. Highly repeated, nanoscale surface features are capable of being formed with inexpensive photolithographic approaches. These repeating surface features are capable of combining properties of surface chemistry (e.g., surface energy) with biological attachment of adhesion/matrix peptides [112,113]. Similar photolithographic approaches have been used to create repeated patterns to generate controlled alternating domains of *N*-(2-aminoethyl)-3-aminopropyl-trimethoxysilane (EDS) and dimethyldichlorosilane (DMS) as a means to control the adsorption of naturally occurring RGD adhesive proteins in serum (especially vitronectin) [101,103]. In this way, a bioactive surface may be generated that uses the natural adhesive proteins in the blood plasma at the time of implant placement. Obviously, there are still multiple issues of bioavailability and biological stability of these peptides that are being worked out but promise holds with such techniques.

The other direction for manipulating biological responses is to create topographical surface features at the nanoscale level on the titanium oxide surface. Relevant nanometer (10^−9^ m) scale features typically means in the range of 1–100 nm in dimension. The interest in this area of research is that the conventional Newtonian properties of materials are very different for a nanomaterial (e.g., increased number of atoms at the surface, surface grain boundaries, enhanced surface energy and surface area, electron delocalization, *etc.*) [48,71–73,114–117]. At the nanoscale level molecular interactions with the surface can be targeted to create specific cell level responses. For instance, work done with nanophase ceramics more than a decade ago demonstrated a specific increase in osteoblast cell adhesion, differentiation and matrix expression on surfaces with a 60 nm grain size or less [118]. If the grain size is 70 nm or greater the specific biological effects were lost. Further studies suggested this effect may be related to protein orientation to the nanophase structures and specifically the mode of orientation of adhesion proteins such as vitronectin to the grain boundaries which in turn alters osteoblast adhesion and shape; both critical to formation of bone [41,116,119–122].

The use of dental implants to replace missing teeth has made rapid progressions over the past twenty five years. Ongoing developments in the area of surface technology are aimed at enhancing tissue/surface interactions and to potentially use the expanding knowledge regarding the immune response, especially the role of the highly complex macrophage family of immune affecter cells. As our knowledge of these complex pathways is incomplete so is our ability to currently predict the biological responses to changes in surface properties. As the disciplines of immunology continues to understand the wound healing process, biomaterials development plays a complementary role as an interdisciplinary approach to developing implant surfaces that mimic and promote accelerated wound healing processes. All of these ongoing developments have a central goal of enhancing patient care.
